# Influence of Pigment Epithelium-Derived Factor on Outcome after Striatal Cerebral Ischemia in the Mouse

**DOI:** 10.1371/journal.pone.0114595

**Published:** 2014-12-03

**Authors:** Marietta Zille, Arina Riabinska, Menderes Yusuf Terzi, Mustafa Balkaya, Vincent Prinz, Bettina Schmerl, Melina Nieminen-Kelhä, Matthias Endres, Peter Vajkoczy, Ana Luisa Pina

**Affiliations:** 1 Department of Experimental Neurology, Center for Stroke Research Berlin, Charite - Universitaetsmedizin Berlin, Berlin, Germany; 2 Department of Neurosurgery, Charite - Universitaetsmedizin Berlin, Berlin, Germany; 3 Department of Neurology, Charite - Universitaetsmedizin Berlin, Berlin, Germany; School of Pharmacy, Texas Tech University HSC, United States of America

## Abstract

We here suggest that pigment epithelium-derived factor (PEDF) does not have an effect on lesion size, behavioral outcome, cell proliferation, or cell death after striatal ischemia in the mouse. PEDF is a neurotrophic factor with neuroprotective, antiangiogenic, and antipermeability effects. It influences self-renewal of neural stem cells and proliferation of microglia. We investigated whether intraventricular infusion of PEDF reduces infarct size and cell death, ameliorates behavioral outcome, and influences cell proliferation in the one-hour middle cerebral artery occlusion (MCAO) mouse model of focal cerebral ischemia. C57Bl6/N mice were implanted with PEDF or artificial cerebrospinal fluid (control) osmotic pumps and subjected to 60-minute MCAO 48 hours after pump implantation. They received daily BrdU injections for 7 days after MCAO in order to investigate cell proliferation. Infarct volumes were determined 24 hours after reperfusion using magnetic resonance imaging. We removed the pumps on day 5 and performed behavioral testing between day 7 and 21. Immunohistochemical staining was performed to determine the effect of PEDF on cell proliferation and cell death. Our model produced an ischemic injury confined solely to striatal damage. We detected no reduction in infarct sizes and cell death in PEDF- vs. CSF-infused MCAO mice. Behavioral outcome and cell proliferation did not differ between the groups. However, we cannot exclude that PEDF might work under different conditions in stroke. Further studies will elucidate the effect of PEDF treatment on cell proliferation and behavioral outcome in moderate to severe ischemic injury in the brain.

## Introduction

Stroke is one of the leading causes of death and disability in the Western societies. Despite the increasing need, the only effective therapy is thrombolysis with recombinant tissue plasminogen activator. However, only 7% of the patients can benefit in a time window of up to 4.5 hours [1].

Neurotrophic factors, including pigment epithelium-derived factor (PEDF), are of increasing interest for therapy. Along with other effects, they prevent initialization of cell death while promoting differentiation of neuronal progenitor cells [2].

The endogenous role of PEDF in the CNS has not yet been completely determined. Its known effects include neurotrophism, neuroprotection, antitumorigenesis, and antiangiogenesis [3]. PEDF is produced in most mammalian tissues, including the brain [4]–[6]. It was found in striatum [7], cerebellum [8], hippocampus and hypothalamus [9] as well as cerebral cortex [9]–[11].

PEDF is neuroprotective after a variety of neuronal injuries [12]. In particular, it prevented neuronal cell death in cell culture and to rescue cultured neurons from glutamate- and 6-hydroxydopamine-induced toxicity [13]–[15]. Intrinsic PEDF protein and mRNA were downregulated after permanent middle cerebral artery occlusion (MCAO) in the rat [16]. In a study using transient MCAO in the rat, gene transfer of PEDF significantly decreased infarct size and edema, attenuated cell death, and reduced inflammation [8].

The mechanisms underlying the neuroprotective effects of PEDF upon ischemic injury in the brain are poorly understood. One possible mechanism might be its role in cell proliferation. In the brain, PEDF promoted self-renewal of adult neuronal stem cells [17]. Similarly, PEDF increased metabolic activity in microglia while blocking proliferation. In astrocyte-microglia co-cultures, PEDF did not change the metabolic activity of astrocytes, but their proliferation was inhibited [18]. PEDF also improved survival of cultured embryonic cerebellar granule cells, whereas there was no effect on cell proliferation [13]. PEDF upregulation induced by mamentine (an NMDA receptor antagonist) increased cell proliferation of hippocampal progenitors. Such an effect was not replicated overexpressing PEDF using an adeno-associated virus [19]. On the other hand, PEDF expression was reduced or absent in glioma [4] and transient PEDF expression decreased the proliferation of cancer cells in the brain [20].

We here investigated whether intraventricular infusion of PEDF reduces infarct size and cell death, ameliorates behavioral outcome, and influences cell proliferation in the one-hour MCAO model in the mouse. We chose the MCAO model because it is well established in our lab and the most commonly used experimental model of focal cerebral ischemia. We implanted mice with PEDF or artificial cerebrospinal fluid (aCSF, control) osmotic pumps and subjected to 60-minute MCAO 48 hours after pump implantation. To investigate cell proliferation, mice received daily BrdU injections for 7 days after MCAO. We also determined infarct size, behavioral outcome, and cell death. Under our experimental conditions, PEDF did not have an effect on lesion size, behavioral outcome, cell proliferation, or cell death upon ischemic injury confined to striatal injury.

## Materials and Methods

### Experimental Animals

Animal experiments were performed in consent with national and international guidelines for the care and use of laboratory animals (Tierschutzgesetz der Bundesrepublik Deutschland, European directive, as well as GV-SOLAS and FELASA guidelines and recommendations for laboratory animal welfare) and approved by an ethics committee (Landesamt für Gesundheit und Soziales, Berlin, Germany, permit number: G 0270/10).

Eight- to eleven-week-old Male C57BL6/N mice were acquired from Charles River Laboratories, Germany. We treated animals with respect to the German Animal Welfare Act and the European Council Directive 86/609/EEC concerning the protection of experimental animals. Animals were housed in groups of 3-6 per cage with a 12/12 hours light/dark cycle controlled environment and given ad libitum access to food and water.

### Experimental Design and Exclusion Criteria

**Figure 1:**
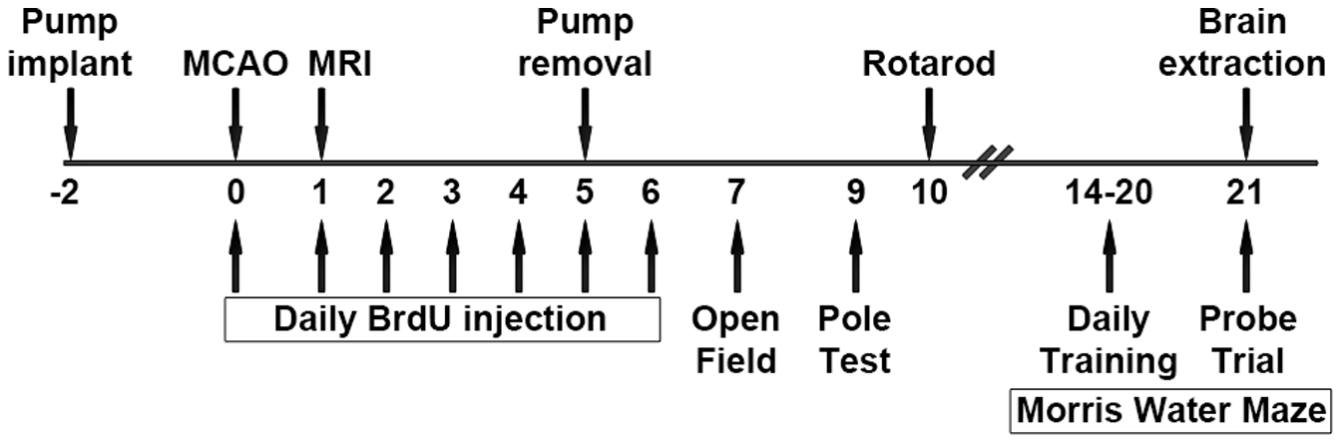
Experimental Design. Mice underwent osmotic pump implantation two days prior to MCAO. Starting at the day of the MCAO, mice received daily intraperitoneal injections of Bromo-deoxy-Uridine (BrdU) for 7 days. 24 hours after reperfusion, T2-weighted magnetic resonance imaging (MRI) was performed. On day 5 after MCAO, osmotic pumps were removed. Animals underwent behavioral testing: Open Field test at day 7, Pole test at day 9, rotarod at day 10, and Morris Water Maze (MWM) with seven days of training prior to probe trial on day 21. Then, animals were sacrificed and brains were extracted for immunohistochemical analysis.

All experimental procedures were performed by investigators blinded to the experimental conditions. Animals were randomly assigned to the experimental groups. The experimental design is depicted in Figure 1.

Twelve mice weighing 19 to 24 g at the time of operation underwent osmotic pump implantation followed by MCAO with an interval of two days. We removed the pumps 7 days after implantation, i.e. 5 days after MCAO. We performed T2-weighed magnetic resonance imaging (MRI) at 24 hours after MCAO. From day 7 to day 21 after MCAO, animals underwent behavioral testing. Besides, from day 0 to day 7 post-MCAO, we injected Bromo-deoxy-Uridine (BrdU) intraperitoneally on a daily basis for immunohistochemical analysis of cell proliferation. Accordingly, we sacrificed the mice and extracted their brains for tissue analysis on day 21 after MCAO.

We chose the behavioral test battery and time points for the tests based on own previous results using these tests in MCAO animals [21] and the literature (for an extensive review on assessing post-stroke behavior in the mouse see Balkaya et al [22]). We used Open Field test on day 7 to evaluate exploratory locomotion and anxiety. Pole test on day 9 and rotarod on day 10 were chosen to assess motor function. Using Morris Water Maze (MWM), we investigated spatial learning and memory. We performed training for MWM for seven days prior to probe trial on day 21.

Two animals were excluded from the experiment because they had no lesion identified on MRI 24 hours after MCAO. One brain of the PEDF group was excluded from the histochemical analysis due to bad tissue quality.

### Osmotic Pump Implantation

We primed micro-osmotic Alzet pumps (model 1007D) under sterile conditions one day before implantation and left them in an incubator at 37.0°C overnight. We anesthetized the animals with a ketamine (Ketavet, Pfizer, Germany, 150 mg/kg) and xylazine (Rompun, Bayer, Germany, 15 mg/kg) mixture dissolved in 8.5 ml of sterile 0.9% saline. Head skin incision was performed exposing the skull.

We implanted the cannula 0.2 mm to the right from Bregma corresponding to the location of the right ventricle. A drop of cyanoacrylate clay (Weicon, Germany) was introduced between the cannula cap and the skull. We slowly moved the cannula down until the cap touched the skull. We left the animals for 5 min to ensure drying of the clay. Then, we carefully sutured the skin above the cannula with a 5–0 polypropilen thread (Ethicon, USA).

Animals received lidocaine gel (Xylocain, AstraZeneca, Germany) locally and 0.5 ml of ringer lactate solution (B Braun Melsungen, Germany) intraperitoneally to substitute the liquid loss. We placed the cage with operated animals onto the 37°C warm bed for 2 hours.

The animals received a total amount of 84±16.8 µl of PEDF (20 µg/ml in CSF) or CSF at a pumping rate of 0.5±0.1 µl/hour.

### Middle Cerebral Artery Occlusion

We induced transient middle cerebral artery occlusion (MCAo) by inserting a silicone rubber-coated 190 µm thick monofilament (Doccol Corporation, Redlands, USA) through an incision in the left common carotid artery into the internal carotid artery as described by Engel and colleagues [23]. Mice were anesthetized with 1.5% Isoflurane in a 2/3 N_2_O and 1/3 O_2_ mixture and kept under anesthesia during the whole operation procedure. After 60 min of ischemia, we re-anesthetized the animals and removed the filament to permit reperfusion.

Mice were treated with Lidocain (Xylocain, AstraZeneca, Germany) as a local anesthesia directly after and around 6 hours after surgery. During surgery and ischemia, we measured their body temperature and kept it constant between 37.0 and 37.5°C with a heating pad. After surgery, the animals were allowed to wake up in a warming cage and kept there for around 2 hours. MCAO was confirmed by observing the animals circling after operation and by MRI at 24 hours.

### Magnetic Resonance Imaging

24 hours after MCAO, mice were scanned in a 7 Tesla Pharmascan 70/16 (Bruker Biospin MRI GmbH, Ettlingen, Germany) equipped with a 16 cm horizontal bore magnet and a 9 mm (inner diameter) shielded gradient (300 Mhz H-resonance frequency, maximum gradient strength 300 mT/m, rise time 80 µs). We carried out data acquisition and image processing with the Bruker software (Paravision 4.0).

For the examination, animals were anesthetized with isoflurane (2.5% for induction, 1.5% for maintenance, Forene, Abbot, Wiesbaden, Germany) in 70% N2O and 30% O2 via a facemask under constant ventilation monitoring (Small Animal Monitoring & Gating System, SA Instruments, Stony Brook, New York, USA). We kept their body temperature constant at 37°C during measurement using a heated circulating water blanket.

For imaging the mouse brain, we used a T2-weighted 2D turbo spin-echo sequence (TR/TE: 4200/36 ms, rare factor 8, 4 averages). Twenty 0.5 mm-hick axial slices over the brain from olfactory bulb to cerebellum were imaged with a field of view of 2.56×2.56 cm and a matrix size of 256×256, resulting in a nominal voxel size of 98 µm×98 µm. The acquisition of the T2-weighted images lasted 6 min 43 s.

### Infarct volumetry and edema assessment

We processed the images acquired during magnetic resonance scanning with ImageJ v1.42 (NIH, Bethesda, USA) and Analyze 5.0 (BIR, Mayo Clinic, USA) software. We outlined ipsilateral and contralateral hemispheres as well as the ischemic infarct represented by hyperintense areas in T2-weighted images where present in each of the 20 slices. To estimate the infarct volume, we applied a correction for edema by calculating the ‘indirect’ infarct volume as the volume of the contralateral hemisphere minus the non-infarcted volume of the ipsilateral hemisphere. In order to assess the contribution of the edema, the percentage volume increase of the ipsilateral compared to the contralateral hemisphere was calculated.

### BrdU injection

Mice received daily intraperitoneal BrdU injections of 50 mg per kg body weight (diluted in 0.9% NaCl-solution) over a period of 7 days as of the day of MCAO.

### Open Field Test

The open field test is comprised of an unfamiliar open field, which is an undifferentiated square of 50×50 cm surrounded by high walls. On day 7 after MCAO, mice were given one trial each in which they were allowed to explore freely for 10 min. We monitored locomotor activity using a video tracking system (VideoMot 2 v.5.68, TSE, Bad Homburg, Germany and black/white CCD Camera, Robert Bosch GmbH, Stuttgart, Germany). We recorded the total distance each mouse traveled and time spent in the open center of 35×35 cm.

### Pole Test

Pole test was performed as previously described [24], with minor modifications. The test apparatus consisted of a vertical steel pole covered with tape (Durapore, 3 M) to create a rough surface. On day 9 after MCAO, we placed the mice head upward on the top of the pole. We recorded the time the mouse took to turn completely head downwards and the total time it took to descend down and reach the floor with its front paws. If the animal was unable to turn completely, we attributed the time to reach the floor to *t* turn, too. We performed the test four times and used the mean of the four trials for statistical analysis.

### Rotarod

We performed Rotarod as previously described [25]. On day 10 after MCAO, we placed the mice on an accelerating rotating rod (from 4 to 40 rpm over 300 s) and their latency to fall was recorded. We performed the test three times and used the mean of the three trials for statistical analysis.

### Morris Water Maze Test

Experiments were performed as described with minor modifications [26]–[28]. A 120-cm-diameter, we filled a 60-cm-high circular swimming pool to a depth of 32 cm with 20°C opaque water. We placed visible cues on the walls of the pool, which remained on their fixed position throughout the whole experiment. A clear Plexiglas platform with a diameter of 11 cm was submerged 1 cm below the water level.

A full experiment had two phases: a place task (learning period) with three trials per day for seven consecutive days (day 14–20 after MCAO) and a probe trial (spatial probe) on the eighth day (day 21 after MCAO). For the place task, we placed the platform near the center of a quadrant and released the mice into the water from one of the three remaining quadrants to search for the platform. If after 90 s an animal did not reach the platform, we guided it to the platform. After reaching the platform, we allowed the animals to remain there for 30 s. The intertrial interval was 1.5 min, and we dried the mice with a towel and put under a heating lamp between each trial to avoid hypothermia.

We recorded escape latencies to find the platform, total distance traveled and swim speed with a computer-based system (VideoMot 2 v.5.68, TSE, Bad Homburg, Germany and black/white CCD Camera, Robert Bosch GmbH, Stuttgart, Germany). We used the mean of the three trials per day for statistical analysis. In the probe trial, we allowed the mice to swim freely for 90 s in the absence of the platform. We measured the time spent in each quadrant and crosses through the location of the former platform.

### Immunohistochemistry

For all immunohistochemistry analyses, animals were anesthetized and perfused transcardially with 0.9% saline, followed by 4% PFA in saline. Brains were extracted and kept in PFA solution for 2 hours, then transferred into 30% sucrose and snap-frozen. 20 µm thick coronal slices were cut using a cryostat (HM560, Microm, Germany) and every second section was preserved. We arranged slices on glass slides (six sections with a distance of 1 mm from Bregma 2.1 to −3.4). We kept the slides at −20°C until used for immunohistochemistry.

For BrdU histochemistry, we washed the cryo sections twice in PBS for 10 min each followed by treatment with 0.6% H_2_O_2_ for 30 min and brief rinsing afterwards. The sections were incubated in a pre-warmed 2 N HCl solution for 30 min at 37°C. We then washed the sections with 0.1 M borate buffer (pH 8.5) for 10 min. After 2× washing with PBS, the sections were incubated in blocking solution (3% donkey serum in PBS/Tween 0,1%) for 1 hour to block unspecific binding of primary anti-BrdU antibody. Thereafter, we incubated the sections with rat anti-BrdU antibody (1∶500, AbD Serotec, OBT 0030) overnight at 4°C. On the next day, after washing, we treated the sections again with blocking solution for 10 min and incubated them with biotinylated donkey anti-rat antibody (1∶250, Jackson ImmunoResearch Laboratories, 712-065-150) for 2 hours at RT. After washing, we incubated the sections with ABC-HRP kit for 2 hour and washed again. The reaction was developed by incubating with DAB for 6–10 min and after washing, the sections were mounted.

For double stainings, we washed the slices twice in PBS and incubated in pre-warmed 2 N HCl solution for 30 min at 37°C. We subsequently neutralized with 0.1 M borate buffer (pH 8.5) for 10 min. The slices were then incubated in blocking solution for 1 hour and stained with rat anti-BrdU antibody (1∶500, AbD Serotec, OBT0030) together with either mouse anti-GFAP antibody (1∶100, Millipore, MAB360), goat anti-Iba1 antibody (1∶100, Abcam, ab5076) or mouse anti-NeuN antibody (1∶50, Millipore, MAB377) primary antibodies overnight at 4°C.

On the next day, after washing, we treated the sections again with blocking solution for 10 min. Then, we incubated them with the appropriate secondary antibodies (all from Jackson ImmunoResearch Laboratories if not otherwise indicated): anti-rat-Cy3 (1∶100, 712-165-153) and either anti-mouse-DyLight488 (1∶50, 715-485-151) or anti-goat-DyLight549 (1∶100, 705-505-003). For BrdU/NeuN double stainings, we used the secondary antibodies anti-rat-DyLight488 (1∶50, 712-485-150) and anti-mouse-DyLight549 (1∶50, 715-505-150). Counterstaining was done with DAPI (1∶100, 5 µg/ml, Sigma Aldrich, D9564).

### TUNEL

To determine the impact of PEDF on cell death, we performed TUNEL using ApopTag kit (Millipore, USA, Catalogue Number: S7165) according to the protocol given. Briefly, we fixed the cryosections in 1% PFA for 10 min at room temperature and postfixed them in ethanol-acetic acid mixture (2∶1) for 5 min in −20°C. After washing in PBS, specimens were shortly (10 s) incubated with equilibration buffer supplied with the kit and afterwards incubated with TdT enzyme solution for 1 hour at 37°C. We then applied stop/wash buffer, washed the specimens and incubated them for 30 min with rhodamine at room temperature. We washed the specimens again in PBS, mounted them with a mounting medium and coverslipped the specimens.

### Fluorescence Microscopy and Image Assessment

Immunohistochemistry stainings were analyzed with a microscope (Olympus BX-61, USA) and Cell^∧^P software. Pictures were taken at 100× magnification, with field size 0.68×0.89 mm. Images were quantitatively analyzed in Cell^∧^P software.

For BrdU, we counted the cells on two slices per animal. For cortex and striatum four fields of view per slice and for subventricular zone (SVZ) and dentate gyrus the whole area was investigated according to the counting method of Weidner and colleagues [29]. For the SVZ, we counted the cells in a distance of 50 µm from the ventricle. For double stainings, we counted the cells in the whole striatum on one slice per animal.

For TUNEL, we counted the cells on ipsilateral and contralateral striatum on four slices with a distance of 1 mm each. We used the whole striatum on each slide for analysis. We divided the number of cells by the area in which we counted the cells to obtain cells/mm^2^. Then, we subtracted the number of cells in contralateral striatum from ipsilateral striatum and calculated the mean for each animal.

### Statistical Analysis

We tested normality using graphical methods and confirmed them by Kolmogorov-Smirnov test. Variance homogeneity was evaluated using Levené test. When data were normally distributed, variance homogeneity was met and two independent groups were investigated, student's t-test was performed. In case of comparing two groups with repeated measures, we performed ANOVA with Box's M test for equality of covariance matrices and Levené test for variance homogeneity. Data are represented as mean ± standard deviation. Differences are considered significant at p<.05. We performed all statistical analyses with SPSS v.19.0.

## Results

### PEDF Treatment Does Not Affect Lesion Size After Striatal Ischemic Injury

Mice underwent aCSF or PEDF pump implantation as well as MCAO two days afterwards. 24 hours after MCAO, we performed T2-weighted MRI. For both lesion volume and edema, data were normally distributed (Kolmogorov-Smirnov test, Z = .743, p = .639 for lesion volume, Z = .599, p = .866 for edema) and variances were homogenous across groups (Levené test, F(1,8) = 1.171, p = .311 for lesion volume, F(1,8) = 2.718, p = .138 for edema).

**Figure 2:**
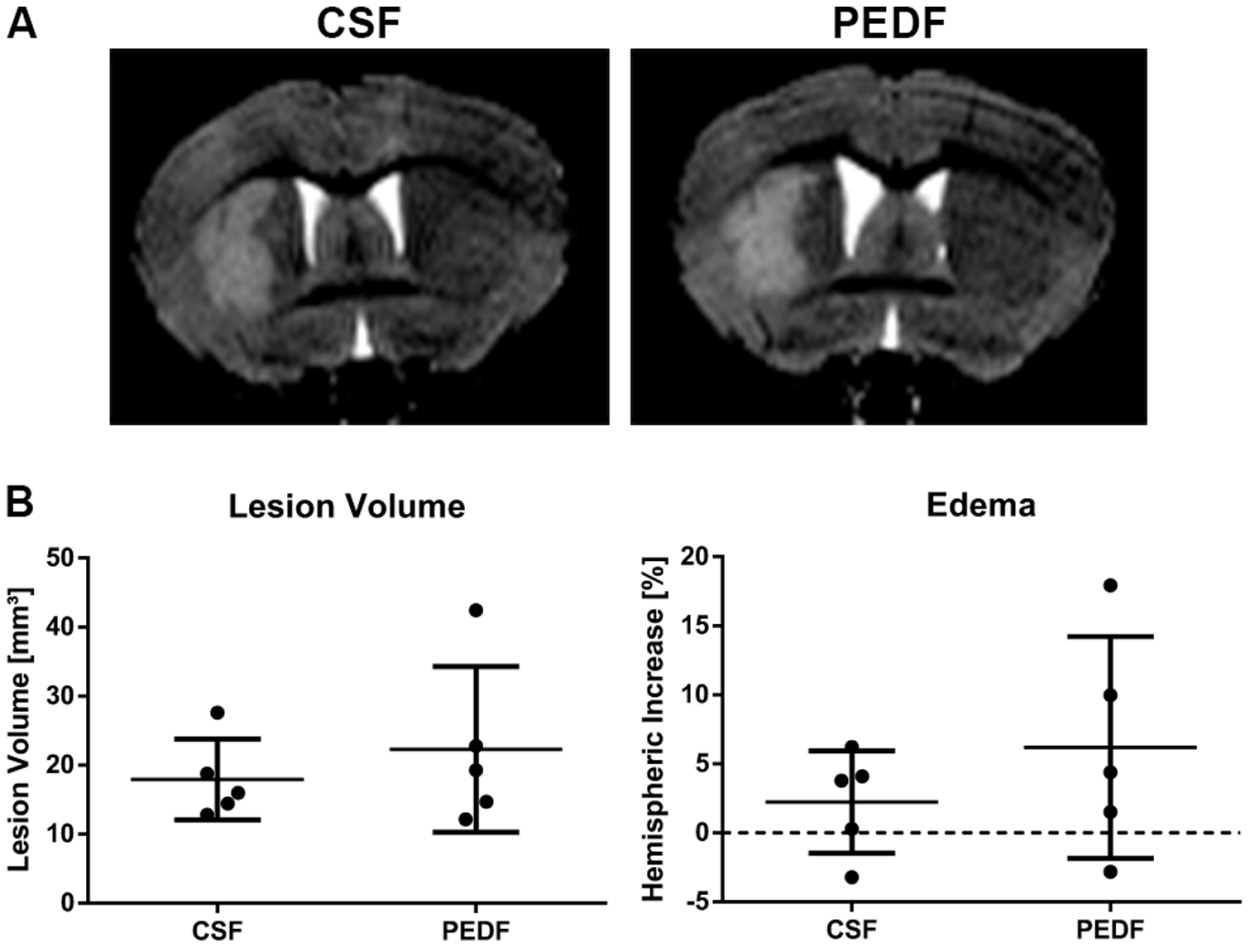
PEDF Treatment Does Not Affect Lesion Size After Striatal Ischemic Injury. Mice underwent T2-weighted MRI at 24 hours after MCAO. (A) Representative T2-weighted images of CSF and PEDF groups are shown. (B) Lesion Volume and edema were not significantly different between both groups. Data is represented as means ± SD. n = 5 per group.

Mean lesion volumes were 17.9±5.8 and 22.3±12.0 mm^3^ for CSF and PEDF (n = 5 per group) respectively. There was no significant difference between the groups (independent t test, t(8) = −.728, p = .487, d = .461). Mean relative edema volumes were 2.24±3.72 and 6.19±8.04% for CSF and PEDF (n = 5 per group) respectively. There was no significant difference between the groups (independent t test, t(8) = −.997, p = .348, d = .631), either (Figure 2).

### PEDF Treatment Does Not Affect Exploratory Locomotion and Anxiety After Striatal Ischemic Injury

To investigate whether PEDF treatment has an effect on behavioral outcome after striatal ischemic injury, we performed four different behavioral tests. First, we evaluated exploratory locomotion and anxiety using Open Field test on day 7.

We measured total distance traveled as well as time spent in the open center of the box. For both parameters, data were normally distributed (Kolmogorov-Smirnov test, Z = .510, p = .957 for total distance traveled, Z = .476, p = .977 for time spent in open center) and variances were homogenous across groups (Levené test, F(1,5) = 2.771, p = .157 for total distance traveled, F(1,8) = 3.992, p = .102 for time spent in open center).

**Figure 3:**
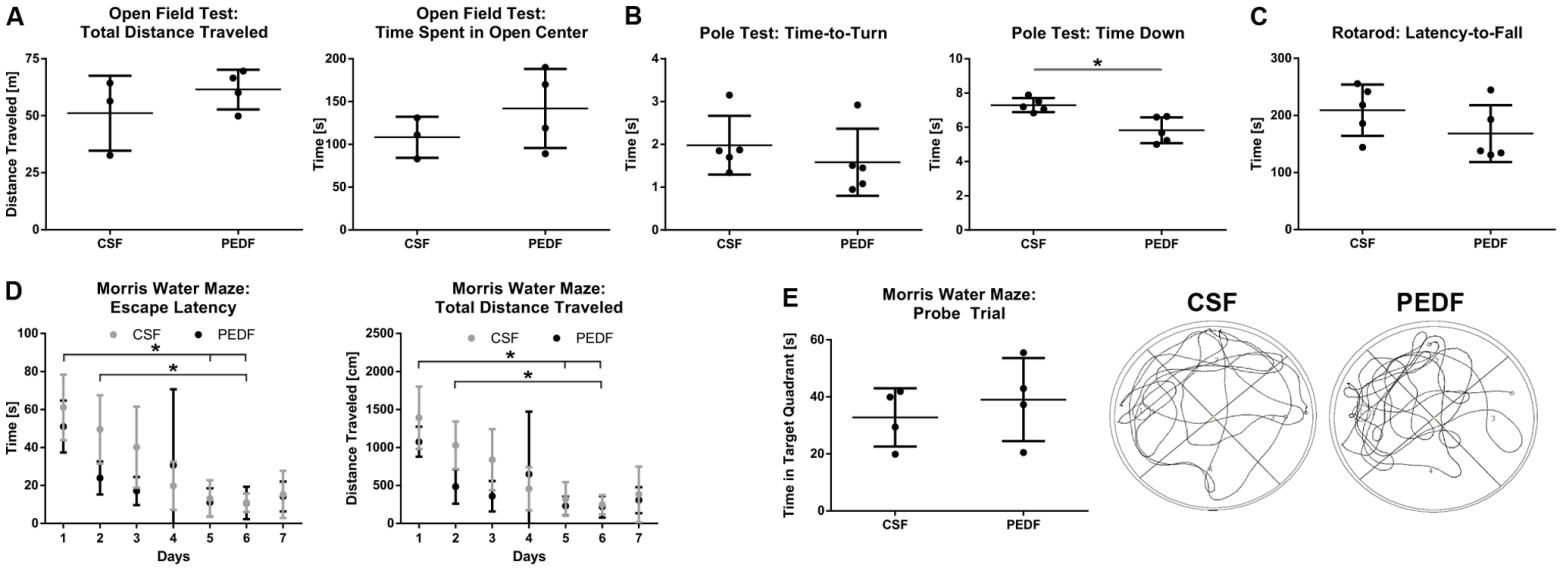
PEDF Treatment Does Not Affect Behavioral Outcome After Striatal Ischemic Injury. (A) Exploratory locomotion and anxiety were evaluated using Open Field test on day 7. Total distances traveled and time spent in the open center were not significantly different between CSF (n = 3) and PEDF (n = 4). (B) Pole test was carried out on day 9 to assess motor function. While the time the mouse took to turn completely head downwards (time-to-turn) was not significantly different across groups, the time to descend down (time down) significantly decreased in the PEDF compared to CSF group (independent t test, t(8) = 3.798, p = .005, d = 2.407, n = 5 per group). (C) Rotarod was also used to investigate the influence of PEDF on motor function after MCAO. On day 9, mean latencies to fall off the rod were not significantly different across groups. (D–E) To investigate spatial learning and memory, Morris Water Maze (MWM) was performed with training for seven days prior to probe trial on day 21. (D) During training, swim speed did not significantly differ between the groups. Escape latencies to reach the platform and total distance traveled were not significantly different between the groups, but between day 1 and day 5 for both PEDF (repeated measures ANOVA, p = .007 for escape latencies and p = .010 for total distance traveled) and CSF (p = .003 for both), but only for CSF between day 1 and 6 (p = .023 for escape latencies and p = .019 for total distance traveled) and day 2 and 6 (p = .038 for escape latencies and p = .040 for total distance traveled). (E) During probe trial, time in target quadrant and swim speed were not significantly different across groups (n = 4 per group). On the right, representative recordings during probe trial for CSF and PEDF groups are shown. Data is represented as means ± SD.

Mean total distances traveled were 52.4±17.2 and 60.6±9.1 m for CSF (n = 3) and PEDF (n = 4), respectively. There was no significant difference between the groups (independent t test, t(5) = −.829, p = .445, d = .596). Mean time spent in the open center was 108.3±24.1 and 142.0±46.3 s for CSF (n = 3) and PEDF (n = 4), respectively. There was no significant difference between the groups (independent t test, t(5) = −1.131, p = .309, d = .912), either (Figure 3A).

Three animals (two from CSF and one from PEDF group) were excluded from this behavioral test being unable to perform the test due to the injury.

### PEDF Treatment Does Not Affect Motor Function After Striatal Ischemic Injury

To assess motor function, we performed Pole test on day 9. We recorded the time the mouse took to turn completely head downwards (time-to-turn) and the total time it took to descend (time down). For both parameters, data were normally distributed (Kolmogorov-Smirnov test, Z = .796, p = .550 for time-to-turn, Z = .680, p = .745 for time down) and variances were homogenous across groups (Levené test, F(1,8) = .051, p = .828 for time-to-turn, F(1,8) = 4.069, p = .078 for time down).

Mean time-to-turn were 2.0±.7 and 1.6±.8 s for CSF and PEDF (n = 5 per group), respectively. There was no significant difference between the groups (independent t test, t(8) = .828 p = .416, d = .543). However, mean time down was significantly decreased in the PEDF compared to CSF group (independent t test, t(8) = 3.798, p = .005, d = 2.407) with mean time down of 7.3±.4 and 5.8±.8 s for CSF and PEDF (n = 5 per group) respectively (Figure 3B).

Another test to investigate the influence of PEDF on motor function after MCAO is Rotarod, which we performed on day 10 recording the latency to fall off the accelerating rod. Data were normally distributed (Kolmogorov-Smirnov test, Z = .680, p = .744) and variances were homogenous across groups (Levené test, F(1,8) = .155, p = .704).

Mean latencies to fall off the rod were 209.1±44.9 and 168.1±49.8 s for CSF and PEDF (n = 5 per group) respectively. There was no significant difference between the groups (independent t test, t(8) = 1.365, p = .210, d = .863) (Figure 3C).

### PEDF Treatment Does Not Affect Spatial Learning and Memory After Striatal Ischemic Injury

To investigate spatial learning and memory, we performed Morris Water Maze (MWM). We performed training for MWM for seven days prior to probe trial on day 21. During training, we measured the escape latency to reach the platform, total distance traveled and swim speed. Swim speed was not significantly different between the groups (data not shown).

For escape latency to reach the platform, the assumption of sphericity was met (Mauchly test, χ^2^(20) = 35.070, p = .054). Variances were homogenous across groups (Levené test, F(1,6) = .449, p = .528 for day 1; F(1,6) = 5.891, p = .051 for day 2; F(1,6) = 1.895, p = .218 for day 3; F(1,6) = 3.373, p = .116 for day 4; F(1,6) = .276, p = .618 for day 5; F(1,6) = .735, p = .424 for day 6; F(1,6) = .372, p = .564 for day 7).

Mean escape latencies were 61.1±17.3 s on day 1, 49.5±18.0 s on day 2, 40.1±21.3 s on day 3, 19.8±12.7 s on day 4, 13.1±9.6 s on day 5, 10.9±4.9 s on day 6, and 15.3±12.4 s on day 7 for CSF, and 51.0±13.6 s on day 1, 23.9±8.6 s on day 2, 17.0±7.4 s on day 3, 30.6±40.0 s on day 4, 11.0±7.5 s on day 5, 10.7±8.5 s on day 6, and 14.2±7.9 s on day 7 for PEDF (n = 4 per group). The omnibus test revealed that there was a significant effect of time (repeated measures ANOVA, F(6,36) = 9.157, p<.001, partial-η^2^ = .604), but not of groups on mean escape latencies (repeated measures ANOVA, F(6,36) = 1.502, p = .205, partial-η^2^ = .200). Bonferroni posthoc analyses revealed a significant difference between day 1 and day 5 for both PEDF (p = .007) and CSF (p = .003), but only for CSF between day 1 and 6 (p = .023) and day 2 and 6 (p = .038) (Figure 3D).

For total distance traveled, the assumption of sphericity was met (Mauchly test, χ^2^(20) = 26.665, p = .272). Variances were homogenous across groups (Levené test, F(1,6) = 2.372, p = .174 for day 1; F(1,6) = 1.793, p = .229 for day 2; F(1,6) = 1.640, p = .248 for day 3; F(1,6) = 2.976, p = .135 for day 4; F(1,6) = 2.741, p = .149 for day 5; F(1,6) = .014, p = .911 for day 6; F(1,6) = 1.567, p = .257 for day 7).

Mean total distances traveled were 1391.1±409.8 m on day 1, 1028.0±312.5bm on day 2, 838.9±401.7 m on day 3, 456.1±283.8 m on day 4, 321.2±221.6 m on day 5, 247.1±127.7 m on day 6, and 382.8±366.5 m on day 7 for CSF, and 1075.4±197.9 m on day 1, 484.5±226.1 m on day 2, 358.6±200.0 m on day 3, 648.3±824.7 m on day 4, 229.1±124.0 m on day 5, 218.9±142.8 m on day 6, and 306.9±170.5 m on day 7 for PEDF (n = 4 per group). The omnibus test revealed that there was a significant effect of time (repeated measures ANOVA, F(6,36) = 9.478, p<.001, partial-η^2^ = .612), but not of groups on mean escape latencies (repeated measures ANOVA, F(6,36) = 1.368, p = .254, partial-η^2^ = .186). Bonferroni posthoc analyses revealed a significant difference between day 1 and day 5 for both PEDF (p = .010) and CSF (p = .003), but only for CSF between day 1 and 6 (p = .019) and day 2 and 6 (p = .040) (Figure 3D).

During probe trial, we recorded time in target quadrant and swim speed. For both parameters, data were normally distributed (Kolmogorov-Smirnov test, Z = .480, p = .975 for time in target quadrant, Z = 1.011, p = .258 for swim speed) and variances were homogenous across groups (Levené test, F(1,6) = .188, p = .680 for time in target quadrant, F(1,6) = 5.295, p = .061 for swim speed).

Mean times in target quadrant were 32.8±10.2 and 39.0±14.5 s for CSF and PEDF (n = 4 per group), respectively. There was no significant difference between the groups (independent t test, t(6) = −.705, p = .507, d = .499, Figure 3E). Mean swim speed was 22.3±4.2 and 20.7±.92 cm/s for CSF and PEDF (n = 4 per group), respectively. There was no significant difference between the groups (independent t test, t(6) = .759, p = .476, d = .537), either. Representative recordings during probe trial for CSF and PEDF groups are shown in Figure 3E.

Two animals (one of each group) were excluded from this test because they were not able to learn the task during the seven days of training.

### PEDF Treatment Does Not Affect Cell Proliferation After Striatal Ischemic Injury

**Figure 4:**
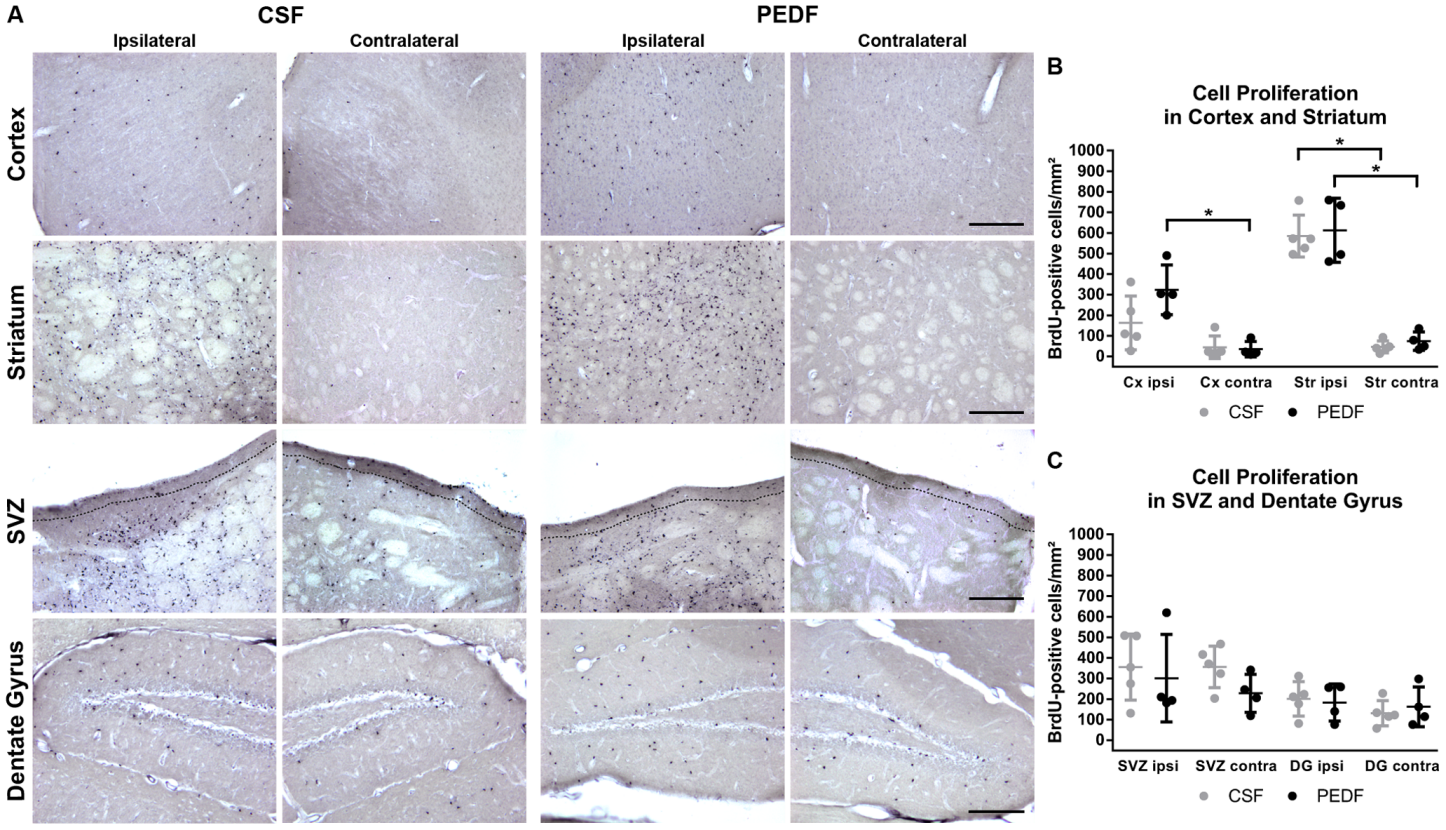
PEDF Treatment Does Not Affect Cell Proliferation After Striatal Ischemic Injury. Mice received daily injections of 50 mg/kg BrdU for seven days starting at the day of MCAO. (A) Representative images of cortex, striatum, subventricular zone (SVZ), and dentate gyrus of CSF and PEDF group. The dotted line in the images of SVZ indicates the area used for counting the cells (distance of 50 µm from the ventricles). Scale bar  = 200 µm. (B–C) Graphs represent the number of BrdU-positive cells/mm^2^ in striatum, cortex, SVZ, and dentate gyrus. We did not find any significant differences between CSF and PEDF groups. (B) We found a significant difference between ipsilateral and contralateral striatum for both PEDF (repeated measures ANOVA, F(1,7) = 76.994, p<0.001) and CSF (F(1,7) = 96.351, p<0.001). However, for cortex, there is a significant difference between ipsilateral and contralateral cortex for PEDF (repeated measures ANOVA, F(1,7) = 14.415, p = 0.007), but not for CSF (F(1,7) = 3.016, p = .124). (C) We did not find any significant differences between ipsilateral and contralateral SVZ and dentate gyrus. Data is represented as means ± SD. n = 5 for CSF, n = 4 for PEDF.

Mice received daily BrdU injections for seven days starting on the day of MCAO. After extraction of the brains at day 21 post-MCAO, we processed the cryosections for BrdU immunohistochemistry. Representative microscopic images of ipsilateral and contralateral striatum, cortex, SVZ, and dentate gyrus for CSF and PEDF group are shown in Figure 4A.

For all regions of interest, the assumption of equality of covariance matrices was met (Box's M test, M = 1.019, M = 1.280, F = .291, p = .832 for striatum; F = .231, p = .875 for cortex; M = 3.620, F = .822, p = .482 for SVZ; and M = 1.812, F = .412, p = .745 for dentate gyrus). The assumption of sphericity was met because there are only two measurement points per group (region of interest on the ipsilateral and contralateral hemisphere).

For all regions of interest and measurement points, variances were homogenous across groups (Levené test, F(1,7) = .931, p = .367 for ipsilateral striatum; F(1,7) = 3.561, p = .101 for contralateral striatum; F(1,7) = .344, p = .576 for ipsilateral cortex; F(1,7) = .173, p = .690 for contralateral cortex; F(1,7) = .051, p = .827 for ipsilateral SVZ; F(1,7) = .349, p = .573 for contralateral SVZ; F(1,7) = .725, p = .423 for ipsilateral dentate gyrus; F(1,7) = .341, p = .577 for contralateral dentate gyrus).

Mean number of BrdU-positive cells/mm^2^ was 46.9±28.7 on contralateral and 585.1±101.9 on ipsilateral striatum for CSF, 74.8±44.8 on contralateral and 612.8±156.1 on ipsilateral striatum for PEDF, 44.4±54.7 on contralateral and 163.4±130.4 on ipsilateral cortex for CSF, 35.6±37.7 on contralateral and 324.2±120.6 on ipsilateral cortex for PEDF, 356.6±100.9 on contralateral and 355.4±160.4 on ipsilateral SVZ for CSF, 228.3±92.6 on contralateral and 301.4±212.9 on ipsilateral SVZ for PEDF, and 131.7±61.1 on contralateral and 201.2±83.3 on ipsilateral dentate gyrus for CSF, 162.7±96.3 on contralateral and 183.1±89.9 on ipsilateral dentate gyrus for PEDF (n = 5 for CSF, n = 4 for PEDF).

For cortex and striatum, the omnibus test revealed that there was a significant effect of hemispheres (repeated measures ANOVA, F(1,7) = 15.970, p = .005, partial-η^2^ = .695 for cortex; F(1,7) = 171.194, p<.001, partial-η^2^ = .961 for striatum), but not of groups on the number of BrdU-positive cells (repeated measures ANOVA, F(1,7) = 2.767, p = .140, partial-η^2^ = .283 for cortex; F(1,7)<.001, p = .997, partial-η^2^<.001 for striatum). Bonferroni posthoc analyses revealed a significant difference between ipsilateral and contralateral striatum for both PEDF (F(1,7) = 76.994, p<0.001) and CSF (F(1,7) = 96.351, p<0.001). However, for cortex, Bonferroni posthoc analyses revealed that there is a significant difference between ipsilateral and contralateral cortex for PEDF (F(1,7) = 14.415, p = 0.007), but not for CSF (F(1,7) = 3.016, p = .124) (Figure 4B).

For SVZ and dentate gyrus, the omnibus test revealed that there is neither a significant difference between hemispheres (repeated measures ANOVA, F(1,7) = .461, p = .519, partial-η^2^ = .062 for SVZ; F(1,7) = 2.940, p = .130, partial-η^2^ = .296 for dentate gyrus) nor between groups on the number of BrdU-positive cells (repeated measures ANOVA, F(1,7) = .493, p = .505, partial-η^2^ = .066 for SVZ; F(1,7) = .875, p = .381, partial-η^2^ = .111 for dentate gyrus) (Figure 4C).

Even though we did not identify any change in overall cell proliferation between the groups, a difference in cell type of proliferating cells was still possible. Therefore, we performed double staining with cell-type specific markers. We did not find any colocalization with NeuN. BrdU-positive cells were positive for GFAP or Iba1.

For both GFAP and Iba1 double staining with BrdU, data were normally distributed (Kolmogorov-Smirnov test, Z = .496, p = .967 for GFAP, Z = .416, p = .995 for Iba1) and variances were homogenous across groups (Levené test, F(1,7) = .111, p = .749 for GFAP, F(1,5) = 1.469, p = .280 for Iba1). Mean number of GFAP^+^/BrdU^+^ cells was 22.7±6.7 and 23.5±10.1 cells/mm^2^ for CSF (n = 5) and PEDF (n = 4), respectively. There was no significant difference between CSF- and PEDF-treated groups (independent t test, t(7) = −.150 p = .885, d = .093). Mean number of Iba1^+^/BrdU^+^ cells was 43.5±18.8 and 34.2±10.9 cells/mm^2^ for CSF (n = 4) and PEDF (n = 3), respectively. Again, there was no significant difference between CSF- and PEDF-treated groups (independent t test, t(5) = −.758 p = .483, d = .605) (Figure S1).

### PEDF Treatment Does Not Affect Cell Death After Striatal Ischemic Injury

We performed TUNEL to assess whether PEDF influences cell death after MCAO. We corrected the number of TUNEL-positive cells in the ipsilateral striatum by subtracting the number of TUNEL-positive cells in the contralateral striatum.

**Figure 5:**
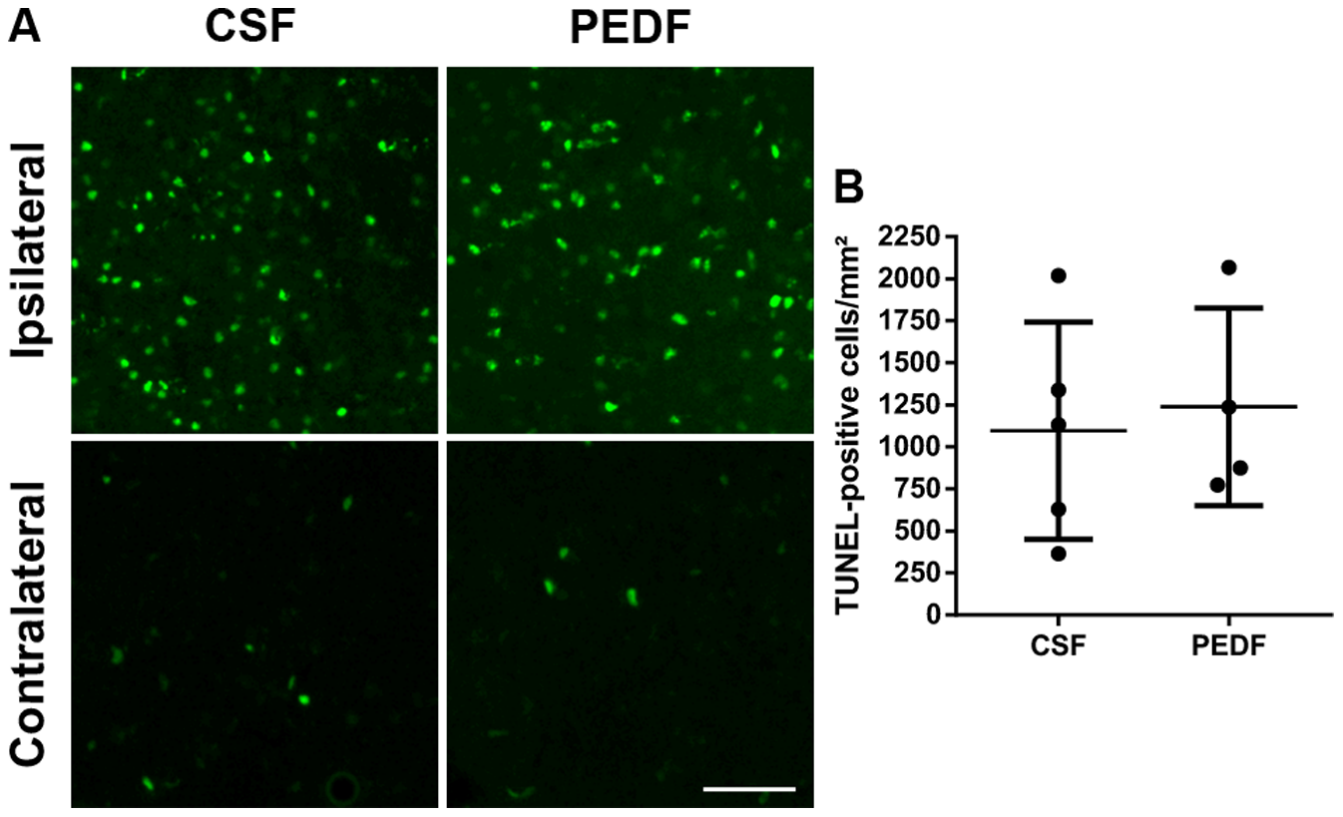
PEDF Treatment Does Not Affect Cell Death After Striatal Ischemic Injury. TUNEL was performed to assess cell death. (A) Representative pictures of ipsilateral and contralateral CSF and PEDF groups are shown. Scale bar  = 100 µm. (B) The number of TUNEL-positive cells in the ipsilateral striatum, corrected by subtraction of the number of TUNEL-positive cells in the contralateral striatum, was not significantly different between both group. Data is represented as means ±SD. n = 5 for CSF, n = 4 for PEDF.

Data were normally distributed (Kolmogorov-Smirnov test, Z = .474, p = .978) and variances were homogenous across groups (Levené test, F(1,7) = .075, p = .792). Mean number of TUNEL-positive cells were 1096±645 and 1238±588 cells/mm^2^ for CSF (n = 5) and PEDF (n = 4), respectively. There was no significant difference between the groups (independent t test, t(7) = −.340, p = .744, d = .230) (Figure 5).

## Discussion

The objective of this study was to investigate whether PEDF can ameliorate behavioral outcome as well as cell death and influences cell proliferation upon focal cerebral ischemia. We demonstrate here that in an ischemic lesion confined to the striatum, PEDF does not exert its previously described protective effects, and has no effect on lesion size, behavioral outcome, cell proliferation, or cell death.

In the present study, we implanted PEDF or CSF osmotic pumps into mice that underwent MCAO two days later and received BrdU injections for 7 days starting at the day of the MCAO. In contrast to a previous study showing that PEDF gene transfer reduces lesion volumes and edema in a model of transient MCAO [8], both were not altered in our study as determined using MRI at 24 hours after reperfusion. However, lesion volumes and percentage of edema were smaller in our model. This might be the reason why PEDF did not exert its protective effects including lesion size reduction, improvement of behavioral outcome as well as reduction of cell proliferation and cell death. Even if PEDF had an effect in this model, the effect might have been too small to be detectable for the methods used in this study.

Another explanation might be that the route, duration, and amount of PEDF administration were not suitable for PEDF to be neuroprotective. In a previous study by Sanagi and co-workers, PEDF overexpression led to protection after MCAO. The authors injected an adenoviral vector containing the PEDF gene directly into the target site [8]. Striatal injection might be superior to intraventricular injections as molecules injected into the cerebrospinal fluid rapidly move into the blood via bulk flow and penetrate brain tissue only poorly [30]. Thus, striatal injection offers the benefit that large molecules such as PEDF do not have to overcome the blood-CSF barrier at the choroid plexus or the blood-brain barrier (BBB).

Interestingly, Pillai and co-workers showed that intravenously injected PEDF overcomes the BBB. They demonstrated a reduction in lesion size along with reduced BBB permeability between 24 hours and 1 week after reperfusion in a rat model of transient focal cerebral ischemia [31]. In addition, Jinnouchi and coworkers have previously shown that PEDF inhibits cold injury-induced brain edema in mice through blocking vascular endothelial growth factor-induced hyperpermeability [32]. Until now, the mechanism of action of PEDF on the BBB after cerebral ischemia remains unclear. We here speculate that we did not sufficiently evoke edema in our model of cerebral ischemia. This might explain why PEDF does not seem to have an effect on behavioral outcome and cell death in this model.

In our study, we decided to use infusion via osmotic pumps because we wanted to directly influence the neurogenic zone. Osmotic pump infusion was previously used successfully for delivery of various trophic factors: Among others, brain-derived neurotrophic factor was applied intraventricularly in a mouse model of Huntington's disease [33] and to the cochlea of deafened guinea pigs [34]. In those studies, neurotrophic factor administration was however performed for a longer period and continued until the termination of the experiment.

We here infused PEDF for only 7 days and ended two weeks prior to final euthanasia. This infusion regime was based on a previous study where Winner and coworkers employed growth factor infusion in a model of PD in rats. They infused epidermal growth factor and fibroblast growth factor-2 for seven consecutive days starting at 8 days after induction of the lesion. The experiment ended either right after the end of growth factor infusion or four weeks later. Using this experimental design, the authors were able to demonstrate that growth factor infusion enhances dopaminergic neurogenesis in the olfactory bulb, but not in the striatum [35].

We chose a pre-MCAO treatment, although not directly applicable to patients, because we wanted to investigate whether PEDF infusion would have an effect at all for stroke recovery. We were not able to perform implantation of the pumps right after MCAO because in a previous experiment, this led to an extremely high mortality.

Another limitation of our study was the use of a single dosage. We want to remark here that it would be beneficial to test different dosages for this experimental design and that we can only report negative findings for this specific dose. However, we here used a dose, which was successful for us in previous experiments (effect on neurogenesis, not published, in preparation). In those previous experiments, we indeed tested three dosages. We decided for the present experimental design to use only the dose that clearly and fully was successful. For this setup, we might have needed a higher concentration for PEDF to be neuroprotective.

To our knowledge, we were the first to investigate the effect of PEDF on behavioral outcome as well as cell proliferation after MCAO. On both, PEDF did not show any effects compared to CSF infusion. We did not use any sham controls to compare whether the behavioral deficits seen in the control group differed from baseline. However, from our previous experience for at least Rotarod and Pole test, we know that the changes we induced were greater than in sham animals [21].

Regarding cell proliferation, intraventricular PEDF compared with vehicle (saline) infusion was previously demonstrated to increase the number of BrdU-positive cells in the SVZ on both hemispheres in healthy animals [17]. In our study, we started PEDF infusion two days prior to MCAO and BrdU injection. Therefore, we might not see the initial increase in BrdU-positive cells. We chose this treatment paradigm because were interested in the effect of PEDF on cell proliferation after MCAO irrespective of its effect on the healthy brain. Here, the number of BrdU-positive cells significantly increased in the ipsilateral compared to contralateral striatum. It was previously shown that cortical injury leads to an increase in cell proliferation in the SVZ. However, whether increased proliferation after brain injury also leads to increased neurogenesis and whether neuronal progenitor cells migrate to the site of injury is not entirely understood until now (for review see Saha et al [36]).

The effect of PEDF on cell death was previously described in models of retinal and in brain ischemia-reperfusion injury. Takita and colleagues demonstrated that PEDF reduced the number of TUNEL-positive cells after intraocular gene transfer [37]. Li and coworkers reported that cell death ameliorated when PEDF was delivered in polylactide-co-glycolide nanospheres [38]. In our study, PEDF did not protect against cell death compared with CSF infusion.

Under our experimental conditions, PEDF does not seem to have an effect in ischemic injury confined to striatal damage only. However, we cannot exclude that PEDF might work under different conditions in stroke. Further studies need to elucidate the effect of PEDF treatment on cell proliferation, cell death and behavioral outcome in moderate to severe ischemic injury in the brain.

## Supporting Information

Figure S1 **PEDF Treatment Does Not Affect Cell Type-Specific Proliferation After Striatal Ischemic Injury.** Double staining of BrdU and cell type specific markers was performed to investigate whether PEDF induces cell proliferation in a certain cell type. Graph represents the number of BrdU^+^/GFAP^+^ as well as BrdU^+^/Iba1^+^ cells/mm^2^. We did not find any significant differences between CSF and PEDF groups. We also did not find any BrdU^+^/NeuN^+^ cells. Data is represented as means ± SD. n = 4–5 for CSF, n = 3–4 for PEDF.
(TIF)
