# Supplementary figures and images for: Influence of Pigment Epithelium-Derived Factor on Outcome after Striatal Cerebral Ischemia in the Mouse
Source: PLoS One. 2014 Dec 3;9(12):e114595. doi: 10.1371/journal.pone.0114595 (PMC4255036; doi:10.1371/journal.pone.0114595)

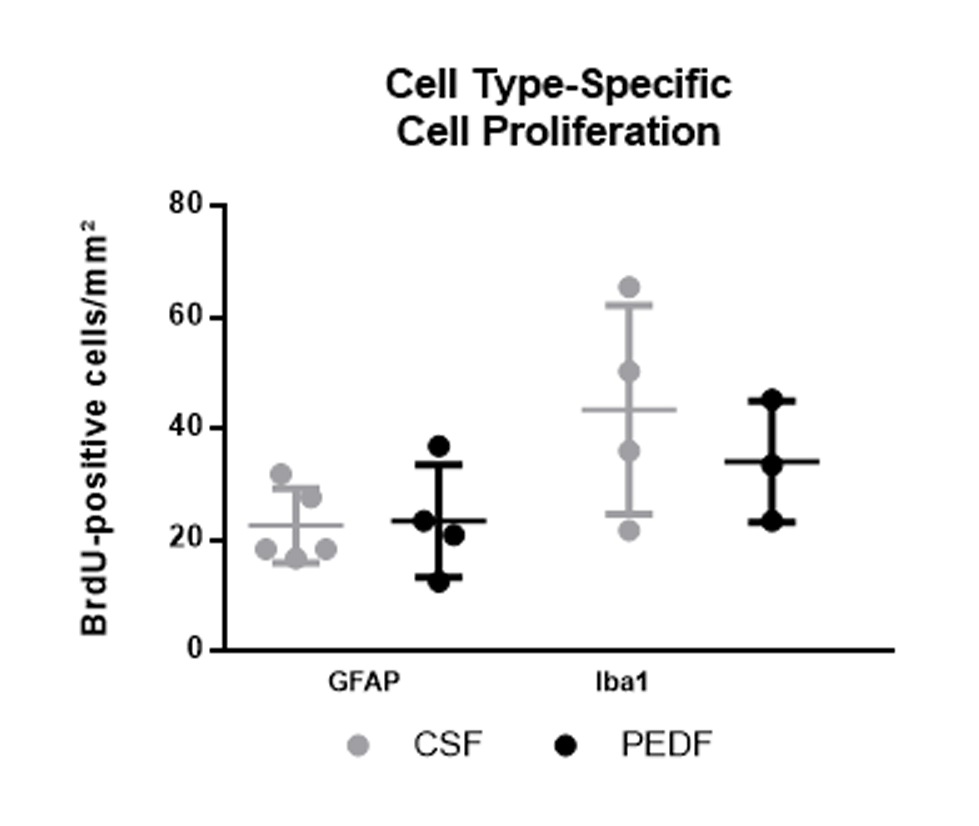

Supplement: Figure S1 — PEDF Treatment Does Not Affect Cell Type-Specific Proliferation After Striatal Ischemic Injury. Double staining of BrdU and cell type specific markers was performed to investigate whether PEDF induces cell proliferation in a certain cell type. Graph represents the number of BrdU+/GFAP+ as well as BrdU+/Iba1+ cells/mm2. We did not find any significant differences between CSF and PEDF groups. We also did not find any BrdU+/NeuN+ cells. Data is represented as means ± SD. n = 4–5 for CSF, n = 3–4 for PEDF. (TIF) [file pone.0114595.s001.tif]
